# Genetic Diversity of Tef [*Eragrostis tef* (Zucc.)Trotter] as Revealed by Microsatellite Markers

**DOI:** 10.1155/2021/6672397

**Published:** 2021-04-22

**Authors:** Mahilet Tadesse, Mulugeta Kebede, Dejene Girma

**Affiliations:** ^1^Adama Science and Technology University, Department of Applied Biology, P. O. Box 1888, Adama, Ethiopia; ^2^Ethiopian Institute of Agricultural Research, Debre Zeit Agricultural Research Center, P. O. Box 32, Debre Zeit, Ethiopia; ^3^Ethiopian Institute of Agricultural Research, National Agricultural Biotechnology Research Center, P.O. Box 249, Holetta, Ethiopia

## Abstract

Genetic variability is the fundamental prerequisite of any crop-breeding program to develop superior cultivars. There are about 350 *Eragrostis*1 species, of which, tef is the only species cultivated for human consumption. Currently, the Ethiopian Biodiversity Institute (EBI) collected over five thousand tef accessions from different geographical regions, diverse in terms of climate and elevation, which are uncharacterized yet. The objective of this study was to evaluate the genetic diversity among 64 tef accessions using 10 selected polymorphic simple sequence repeats (SSRs) markers. A total of 314 alleles were detected with an average of 14.5 alleles per locus and amplicon size ranged from 90 bp–320 bp. The mean value of polymorphic information content (PIC) was 0.87, appearing polymorphic for all loci. The lowest Fst value (0.05) was recorded among the studied tef populations. The mean value of major allele frequency and the number of effective alleles were 0.33 and 3.32, respectively. The mean value of gene flow (Nm) and Shannon's information index (I) was 4.74 and 1.65, respectively. The observed (Ho) and expected (He) heterozygosities varied from 0.34 to 0.56 and from 0.58 to 0.76, respectively. The cluster analysis has grouped the 64 tef accessions into three distinct clusters based on their similarity. The PCoA analysis showed that clustering is basing on the geographical origin of accessions. Analysis of molecular variance revealed 56%, 39% and 5% of the total variation due to variation within populations, among individuals and among populations, respectively. Structure bar-plot also inferred three gene pools, but with high level of admixtures. Thus, the present study shows that the identified tef accessions could be of great interest for the initiation of a planned breeding and conservation programs.

## 1. Introduction

Tef [*Eragrostis tef* (Zucc.) Trotter] is an indigenous cereal crop of great cultural and economic importance in Ethiopia. It belongs to the grass family *Poaceae*, subfamily *Eragrostoideae*, tribe *Eragrostae*, and genus *Eragrostis*. As one of the biggest genus in the grass family, the genus *Eragrostis* contains over 350 species [1]. However, tef is the only species cultivated for human conception [2]. It is an allotetraploid species with a base chromosome number of 10 (2*n* = 4*x* = 40). Cytogenetic studies on tef reported that the genome size of tef is 672 Mbp as described by Cannarozzi et al. [3] and 622 Mbp by Van Buren et al. [4]. Tef is a nutritionally rich cereal crop and contains essential and important nutrients, carbohydrates, protein, fat, fiber, and minerals [5] and has additional health benefits including that the seeds are free from gluten [6]. Some minerals like iron content were significantly higher in tef than in bread wheat [7]. Tef is widely grown across all regions of Ethiopia on a wide range of soil conditions from water-logged vertisols to moisture-stressed, drought-prone soils [8]. Tef is a source of food and income for about 50 million people annually. Over 6 million smallholder farmers in Ethiopia have cultivated it. While its health benefits and nutrition contents, tef is now growing in different countries as food and forage grass including United States, Israel, the Netherlands, Spain, South Africa, India, Australia, and Kenya [8, 9]. Comparatively to other cereal crops, the productivity of tef is significantly low (CSA, 2016). Shattering, the low genetic potential of the local varieties, lodging, and poor agronomic management practices are the major yield-limiting factors [10]. The wealth of diversity in the species offers ample opportunities for genetic improvement of the crop to develop varieties suitable for different agro-ecologies, cropping systems, and purposes. Because of this fact, efforts have been made in the past to assess and quantify the genetic diversity in the tef germplasm collections using different approaches [11]. Molecular markers are short sections of DNA that differ between varieties and thus can be used for the identification of a germplasm by a specific pattern of polymorphisms, to assess diversity and to determine relationships. DNA-based markers provide a very effective and reliable tool for measuring genetic diversity in crop germplasm and studying evolutionary relationships. The molecular markers are superior to both morphological and biochemical markers because they are relatively abundant throughout the genome, independent of environmental conditions, and can be detected at any development stage of the plant [12]. Several studies have been done to examine the diversity in tef germplasm using morphological and agronomic traits. Some of the studies are Assefa et al. [13–17], Adnew et al. [18], Admas and Belay [19] Ayalew et al. [20], Shiferaw et al. [21], and Plaza-Wuthrich et al. [22]. However, few attempts have been undertaken to study the diversity using DNA markers. Ayele [23] by using AFLP markers and Bai et al. [24] by using RAPD markers reported genetic similarity coefficients of 85–90% and 84–96%, which indicated a high level of genetic similarity of the tested genotypes. Previous research [25–31] has shown the usefulness of SSR markers in genetic diversity analysis and in establishing a genetic relationship among tef germplasm. SSR markers are particularly useful since they are codominant, high reproducibility, and polymorphic, have low operational cost and locus-specificity, and revealing of high allelic diversity. To that effect, they have been used extensively in tef and other crops for various breeding and diversity studies [32]. Hence, the objective of this study was to estimate the pattern and level of genetic diversity and relatedness among 64 tef accessions using polymorphic simple sequence repeat (SSR) markers. The study will provide information on the status of genetic variation for further improvement and conservation.

## 2. Materials and Methods

### 2.1. Plant Materials

A total of 64 tef accessions representing nine populations (North Shewa, North Gondar, South Wollo, West Gojjam, Tigray Central Zone, Tigray West Zone, Kembata Tembaro, East Wellega, and Illubabor) were used in this study as shown in (Table 1). These tef accessions were collected by the Ethiopian Biodiversity Institute from tef growing areas of Ethiopia. The study was carried out at Holetta National Agricultural Biotechnology Research Center (NABRC).

### 2.2. DNA Extraction

Twenty-day-old plants grown in a greenhouse and fresh leaves were collected from each seedling. The DNA was extracted using a modified cetyl trimethyl ammonium bromide (CTAB) method [33]. The quality of the DNA was determined by running on 1% agarose gel with 1× TAE buffer (Trizma base with EDTA and boric acid; the pH was adjusted to 8.0 with NaOH) at 100 V for 30 minutes. The quantity (concentration) of the DNA was determined using a Nanodrop spectrophotometer (ND-8000, Thermo Scientific). The molecular weight of each amplified product was estimated by comparing the DNA bands with the high-density standard lambda DNA ladder. The patterns of the amplified product were photographed by a UV transsiluminator lamp (Bio-Doc Imaging System). The DNA was diluted to 50 ng using TE buffer and stored at 4 °C for further PCR work.

### 2.3. Selection of SSR Markers

A total of 15 SSRs markers were used for initial screening developed by Zeid et al. [34]. Out of which, 10 SSRs primers were selected for final diversity analysis based on their level of polymorphism and specificity to the target loci (Table 2).

### 2.4. PCR Amplification

Polymerase chain reaction (PCR) based SSR marker system was applied to investigate the genetic relationships among the sixty-four accessions of tef. PCR amplification was carried out in 96-well plates (Eppendorf Master Cycler Pro S) as per the protocol suggested by Williams et al. [35]. Total PCR reaction was optimized to be 12.5 *μ*l, and this included 6.25 *μ*l 1× One Taq Master Mix (New England Bio Labs), 1 *μ*l of each forward and reverse primers, 0.25 *μ*l Dimethyl-sulfoxide (DMSO, Fisher Scientific), 2 *μ*l nuclease-free water, and 2 *μ*l 50 ng template DNA, respectively. Amplification temperatures were programmed to initial denaturation at 94°C for 3 min followed by 35 cycles of denaturation at 94°C for 1 minute, annealing temperature varies based on the individual primer requirement for 1 minute, primer extension at 72°C for 2 minutes followed by a final extension at 72°C for 10 minutes, and holding temperature at 4°C. The band separation was done by running the PCR products on a 3% agarose gel at 100 V for 2 hours in 1% TAE (Tris-acetate-EDTA) buffer along with a 50 bp DNA ladder. The patterns of the amplified product were photographed by UV transsiluminator lamp (Bio-Doc Imaging System), and the data were saved for further analysis.

### 2.5. Data Analysis

The clear and reproducible alleles amplified by each SSR were scored according to their fragment size (bp) using the PyElph version 1.4 software package [36]. Data from all entries were summarized and converted into a suitable format for various analyses. The number of alleles (Na), number of effective alleles (Ne), Shannon's Information Index (I), gene flow (Nm), observed heterozygosity (Ho), expected heterozygosity (He), analysis of molecular variance (AMOVA), and Hardy-Weinberg equilibrium (HWE) over the entire populations were determined using GenAlEx software version 6.502 [37]. Locus-based diversity indices including major allele frequency (MAF), gene diversity (H), polymorphic information content (PIC), and fixation index (F) were computed using Power marker version 3.25 software [38]. To examine the genetic relationship between the different accessions, a genetic dissimilarity matrix was calculated using Jaccard's formula, and the Unweighted Pair Group Method with Arithmetic Mean (UPGMA) based Neighbor-Joining tree and hieratical clustering analysis was carried out based on population and individual accessions using DARwin version 6.0 [39]. The structure software ver. 2.3.4 based on Bayesian algorithm was applied to determine population structure and admixture pattern [40]. To estimate the true number of population cluster (*K*), a burn-in period of 100,000 was used in each run, and data were collected over 200,000 Markov Chain Monte Carlo (MCMC) replications for *K* = 1 to *K* = 10 using 20 iterations for each *K*. The optimum *K* value was predicted following the simulation system described by Evanno et al. [41] through the web-based STRUCTURE HARVESTER version 0.6.92 [42]. The bar plot for the maximum *K* was determined using Clumpak beta version [43].

## 3. Results and Discussion

### 3.1. SSR Polymorphism

The present investigation revealed high geneticvariation among the studied tef accessions. These accessions were collected from different agroecology of tef growing areas. The study of genetic diversity in any research work is essential as it serves as baseline information for improvement, conservation approaches, and breeding programs. Applying different modern techniques helps in the development of potential verities that are suitable and flexible to climate change and emerging issues that are getting global attention [44]. In this study, 15 SSR markers were screened, out of which, 10 SSR markers were found to be polymorphic and suitable for diversity analysis. The use of SSRs for tef diversity study is very crucial as it provides accurate and unbiased assessment and reveals exhaustive information on the genetic divergence of the accessions [31]. A total of 314 alleles were amplified with an average of 14.5 alleles per locus. The PIC values ranged from 0.61 for the CNLTs5 locus to 0.90 for CNLTs6. The number of amplified markers per SSR primers varied from 12 to 17 with maximum number of alleles (17) being amplified by the primer CNLTs6 and CNLTs25.

### 3.2. Population Genetic Differentiation

Analysis of molecular variance (AMOVA) showed significant differences among and within the populations under assayed. The highest proportion of 56% of the variation was attributed to genetic variability among individuals, while 39% was due to variation among individuals within the same population. In contrast, a smaller portion of 5% of the total variation was among populations (Table 3). Genetic differentiation among the populations was low (FST = 0.05), with a moderate effect of nonrandom mating within the populations (FIS = 0.41). The low (0.05) genetic differentiation among populations was due to the high variability within the populations, which could be due to gene flow. The extensive migration of genes through hybridization of the landraces through developing improved tef varieties could be one of the causes of the current low level of genetic differentiation among populations, and on the other way, the farmers by carelessly handling there could be mixing of the improve varieties with landraces and can be considered as accessions back [45]. Variation of a similar pattern as observed in this study among tef germplasm has been reported in a previous study [28]. In a study, which involved landraces, accessions, and improved varieties collected from different diverse tef growing zones in Ethiopia, 63% of the total variation was attributed to variation within the genotypes. Finding as described by Jifar et al. [31], most genotypes from germplasm accessions had high gene diversity due to more chances of coevolving in nature than those under artificial selection. The breeding lines, however, had the lowest values of all genetic parameters may due to artificial selection towards homogenous populations which drastically reduces the diversity.

### 3.3. Genetic Distance between Populations

The pair-wise population Nei's unbiased the highest genetic distance ranged from 0.540 between population North Gondar and North Shewa accessions to 0.478 between populations of Kembata Tembaro and North Shewa. The next highest genetic distance was observed between populations of Tigray West zone and Kembata Tembaro (0.372) and between the population of West Gojjam and South Wollo (0.341) and followed by Illubabor and West Gojjam (0.325) and East Wellega and North Shewa (0.316). The pair-wise genetic differentiation of low FST value (0.031) was scoured between Kembata Tembaro and East Wellega and followed by 0.051 between East Wellega and North Gondar populations. To explain the properties of subdivided populations, the magnitude of differentiation between and within populations can be quantified using *F* statistics (Fit, Fis, and Fst) also known as fixation indexes (Fst), which is a means to evaluate the population differentiation due to genetic structure. The low Fst value (smaller genetic differentiation) implies that there is a high frequency of identical alleles among accessions [46]. The pair-wise genetic differentiation of moderate FST value accounts for 94% of the total FST values from 0.061 to 0.135 between the populations. The larger FST value was 0.15, which was recorded between Tigray West Zone and South Wollo as showed (Table 4).

### 3.4. Principal Coordinate Analysis

**Figure 1:**
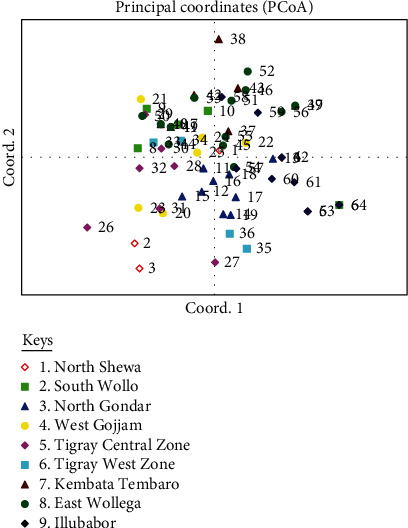
Principal coordinates analysis (PCoA) biplot showing the clustering pattern of 64 tef from the nine populations.

Principal coordinate analysis (PCoA) showed that the first three principal coordinates accounted for about 30.72% of the genetic variation present in the SSR molecular data derived from the 64 accessions used in the study. The first, second, and third principal coordinates explained about 13.13%, 9.20%, and 8.39% of the total variation, respectively. The PCoA analysis in the two-dimensional plot displayed in (Figure 1) showed that accessions from different collection sites often clustered together, showing possible gene flow. A similar finding was also reported by Fikre et al. [30]. There was no separate group formed by a single population possibly because of different factors that favor for intermixing of the germplasm including gene flow and hybridization. The result comes up with the NJ dendrogram, in that there was no unique clustering among accessions from a similar population. The PCoA further supports the previous finding by [25, 47] where mixed clustering was observed among accessions from the different origins of populations. In some cases, accessions of the same population such as East Wollega and North Wollo formed subcluster in the major groups. Even if most of the accessions are forming a subcluster in their specific groups; but there was no separate group formed by a single population. As described on the PCoA biplot, some accession (2, 3, 26, and 38) obtained from populations 1, 5, and 7 were found distantly from the central axis. Therefore, such kinds of accessions are highly recommendable for future. The finding of this study highlighted the need for exploitation of the investigated tef genetic resources to speed up the tef breeding program. Accordingly, the convectional tef varietal refinement efforts should be supported by modern molecular tools and scientific techniques.

### 3.5. Cluster Analysis and Population Structure

**Figure 2:**
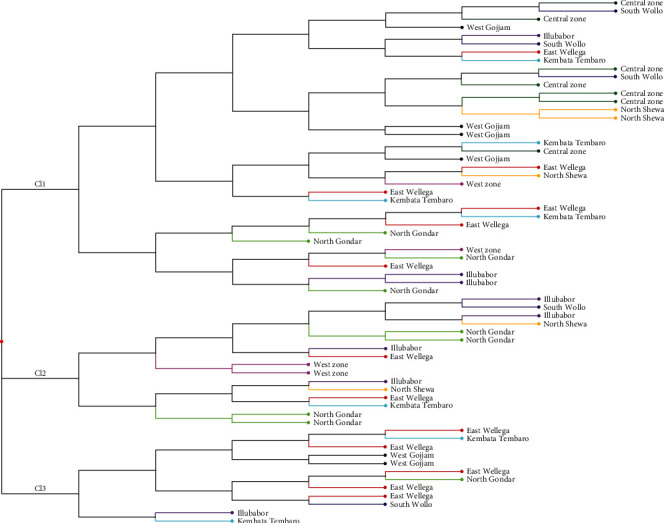
Unweighted neighbor-joining (NJ) dendrogram showing a genetic relationship of 64 tef accessions using 10 SSR markers.

Three major clusters were extracted from all the sixty-four accessions containing 56.25%, 25%, and 18.75% of the total accessions in clusters I, II, and III, respectively. All the clusters were further grouped into two subclusters (Figure 2). The maximum number of populations were included in cluster I having 9 populations and the minimum number in cluster III having 6 populations. Cluster I is composed of accessions from Tigray Central Zone, South Wollo, Illubabor, East Wellega, West Gojjam, Kembata Tembaro, North Shewa, Tigray West Zone, and North Gondar, of which 34. 20% of the total population was from Tigray Central Zone and East Wellega. The clustering pattern indicated the existence of a significant amount of variability among the tef accessions. In the same way, the subclustering of cluster II resulted in two groups composed of six and five populations from Illubabor, South Wollo, North Gondar, North Shewa, Tigray West Zone, East Wellega, and Kembata Tembaro. Likewise, the third cluster contained accessions from East Wellega, West Gojjam, Kembata Tembaro, South Wollo, and Illubabor. The patterns of the grouping of populations across all clusters clearly showed the existence of genetic material independent events of evolutionary forces. Further, the independent events of evolutionary forces such as mutation, selection, genetic drift, and germplasm exchange might separate them into related but different gene pools [48, 49]. The clusters contributing maximum to the divergence were given greater emphasis for deciding the type of cluster for further selection and the choice of the parents of hybridization.

**Figure 3:**
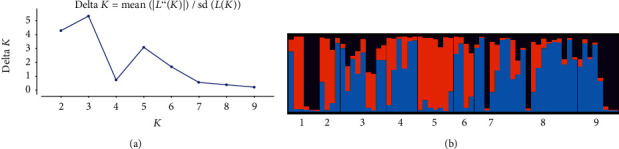
Inferred population structure of 64 tef accessions (a) delta *K* value. (b) Structure bar plot showing the estimated proportion of membership in three clusters as calculated by structure analysis.

The long-standing concern in population genetics is the identification of genetically homogeneous groups of individuals. In this regard, a recent Bayesian algorithm implemented in the software STRUCTURE allows the identification of such groups [42]. The algorithm allows estimating the true number of clusters (*K*) in a sample of individuals included in the study. The analysis revealed that maximum delta *K* (Δ*K*) (which is a good indicator of the true number of clusters) becomes a peak at delta *K*. In this study, the delta *K* value was highest at *K* = 3 (Figure 3(a)) which suggests that 64 diverse accessions of tef can be divided into three subgroups (Figure 3(b)). The number of subgroupings was determined from the delta *K* model described by Evanno et al. [41]. Based on the *K* value, the Clumpak result (bar plot) showed wide admixtures, as a result, there was no clear geographic origin-based structuring of populations (Figure 3(b)). This indicates that the SSR markers we have employed are very powerful to show the genetic relationship among the studied tef accessions.

## 4. Conclusions

High genetic diversity was observed among the tested accessions. The current study highlighted the existence of high levels of diversity among 64 tef accessions, which are suitable for the improvement of potential varieties. SSR markers provide unbiased and sufficient information in the identification and confirmation of tef accessions, as a result, the polymorphism detected among the accessions can be used in breeding programs to exploit the use of genetic resources. Most of the studied populations which had high values of polymorphic information content, gene diversity, and Shannon diversity index could be important to capture adequate information on the diversity state of the germplasm accessions in the Gene Bank. It also indicates the need for giving equal emphasis to all tef growing areas of Ethiopia during germplasm collection and experiment investigation. The dendrogram constructed to identify the genetic similarities among these accessions showed that accessions from the same regions were found to cluster mostly together implying a correlation between molecular groupings and their source of collection. The hierarchical clustering and the population structure analysis assembled the tef accessions under three groups. Hence, the selection of parents must be based on the wider intercluster distance and superior mean performance. As a result, the polymorphism detected among the accessions can be used in tef breeding programs for developing superior tef varieties.

## Figures and Tables

**Table 1:** Passport data of the tested accessions and their geographical origins.

Accession	Local name	Zone	Woreda/district	Altitude (m.a.s.l.)
15309	Tef	North Shewa	Ataye (Efeson)	1443.00
236957	Magna	North Shewa	Minjarna Shenkora	1700.00
236959	Magna	North Shewa	Minjarna Shenkora	1750.00
236960	Tekur tef	North Shewa	Minjarna Shenkora	1780.00
236961	Tikur tef	North Shewa	Minjarna Shenkora	2020.00
243493	Tikur tef	South Wollo	Tenta	2060.00
243502	Ayiro tef	South Wello	Ambasel	1750.00
243507	Sergenga tef	North Wello	Habru	2020.00
243508	Manga tef	North Wello	Guba Lafto	1835.00
243509	Fenkilew	North Wello	Guba Lafto	1835.00
243529	Key tef	North Gondar	Addi Arkay	1310.00
243535	Nech tef	North Gondar	Gondar zuria	1920.00
243536	Sergegna	North Gondar	Gondar zuria	1920.00
243537	Key Fesho tef	North Gondar	Gondar zuria	1920.00
243538	Nech tef	North Gondar	Gondar zuria	1920.00
243539	Key tef	North Gondar	Gondar zuria	2050.00
243540	Nechtef	North Gondar	Gondar zuria	2050.00
243544	Key tef	North Gondar	Gondar zuria	2050.00
243545	Deyabo tef	North Gondar	Gondar zuria	2050.00
243547	Nech tef	West Gojam	Bahirdar zuria	1870.00
243548	Nech tef	West Gojam	Bahirdar zuria	1870.00
243549	Key tef/Ansharo	West Gojam	Bahirdar zuria	1870.00
243550	Key laba tef	West Gojam	Bahirdar zuria	1870.00
243551	Sergegna	West Gojam	Bahirdar zuria	2070.00
243553	Key tef	West Gojam	Bahirdar zuria	1815.00
243512	Tseada tef	Tigray Central Zone	Kola Temben	1990.00
243517	Keyih tef	Tigray Central Zone	Kola Temben	1990.00
243518	Gofgafe tef	Tigray Central Zone	Kola Temben	1990.00
243519	Tseads Dalga	Tigray Central Zone	Kola Temben	1990.00
243520	Murenayi	Tigray Central Zone	Kola Temben	1990.00
243521	Keyih tef	Tigray Central Zone	Kola Temben	2010.00
243524	Tseas tef	Tigray Central Zone	Adwa	2030.00
243525	Sergen tef	Tigray West Zone	Tselemti	1260.00
243526	Tseads tef	Tigray West Zone	Tselemti	1260.00
243527	Tseada tef	Tigray West Zone	Tselemti	1230.00
243528	Sergen tef	Tigray West Zone	Tselemti	1230.00
244789	Dalesha	Kembata Tembaro	Alaba	1773.00
244790	Dorta	Kembata Tembaro	Tembaro	1764.00
244791	Mitazu and Dorta	Kembata Tembaro	Tembaro	1808.00
244792	Metazo	Kembata Tembaro	Kachabira	1717.00
244793	Sergegna	Kembata Tembaro	Kachabira	1714.00
244794	Yemushe	Kembata Tembaro	Kachabira	1714.00
244796	Dalesha	Kembata Tembaro	Alaba	1773.00
244814	Xafi	East Wellega	Bilaseyo	1520.00
244815	Taffee	East Wellega	Bilaseyo	1976.00
244816	Taffee	East Wellega	Bilaseyo	1946.00
244817	Tafii Dima and Adi	East Wellega	Bilaseyo	1946.00
244818	Tafii Adi	East Wellega	Bilaseyo	1973.00
244821	Taffee Adi	East Wellega	Bilaseyo	1952.00
244822	Taffee Dima	East Wellega	Bilaseyo	1910.00
244850	Taffee Adi	East Wellega	Amurujarte	1916.00
244851	Taffee Dima	East Wellega	Amurujarte	1919.00
244852	Taffee Adi	East Wellega	Amurujarte	1876.00
244853	Taffee Adi	East Wellega	Amurujarte	2063.00
244854	Taffee Dima	East Wellega	Gidakiremu	2042.00
244856	Taffee Adi	East Wellega	Gidakiremu	2057.00
244876	Taffee Adi	Illubabor	Bedele	1984.00
244877	Taffee Adi	Illubabor	Bedele	1985.00
244878	Taffee Dima	Illubabor	Bedele	1925.00
244879	Taffee Adi	Illubabor	Bedele	1915.00
244880	Taffee Adi	Illubabor	Bedele	1887.00
244881	Taffee Adi	Illubabor	Bedele	1899.00
244882	Taffee Gomejen	Illubabor	Gechi	1903.00
244883	Taffee Adi	Illubabor	Gechi	1900.00

(Source: Ethiopian Biodiversity Institute (EBI)).

**Table 2:** Locus name, expected size, annealing temperature, and sequence of the 10 selected microsatellite primers.

Primer	Expected size [bp]	Annealing T [°C]	Forward primer	Reverse primer
CNLTs2	160-260	58.2	CAGCAGGGAGAGAGAGGAGA	GGGGTCAAGTTATTGCTTAGAGAA
CNLTs5	190-310	57.2	CCCAAAGTGATGCAAAAACA	TAGATAGAGACACAGACACACACA
CNLTs6	90-260	58.2	AATTCGCAGCTGATCTACGC	CTCGTCGATATACGTGCAAAA
CNLTs19	180-300	58.2	CATTTCTTGCTGCTGGATCA	AGTATGGTGGCCTTGGTGAG
CNLTs22	100-260	55.7	CATGCTCGTTCAGAGTCCAA	GGGGGATCTAGGAGAGAGAGA
CNLTs24	135-180	55.7	GAGAGCGGTTTTGTCCTACG	GGAATAGGGAGGCGAGGTAG
CNLTs25	150-315	57.2	TTGGAATGAGATGGCATTTG	GAAGCGGGGTAAGATTTGAA
CNLTs458	130-250	59.1	AACAAGAACCACACAACA	AAAGGAACCCACAGGGGTAA
CNLTs461	220-320	63.9	GTCTTGATGGTGGCGGAATAG	TCATCATCCTGCTCGAATCA
CNLTs463	190-260	59.1	TGCTAGGATGGTCCTGTTGAG	AGCACCAAATCCCTATGCAC

**Table 3:** Analysis of molecular variance (AMOVA) based on standard permutation across the full data set of tef accessions collected from different geographical origins.

Source of variation	Df	SS	MS	Estimated genetic variance%	*P* value	*F* statistics
Among populations	8	68.36	8.539	0.210 5	0.001	Fst = 0.05
Among individuals	55	308.380	5.607	1.632 39	0.001	Fis = 0.41
Within population	64	150.000	2.344	2.344 56	0.001	Fit = 0.44
Total	127	526.695		4.185 100		Nm = 4.742

Df: degree of freedom; SS: sum of squares; MS: mean squares.

**Table 4:** Pairwise Nei's unbiased genetic distance (upper diagonal) and pairwise FST (lower diagonal).

	NS	SW	NG	WG	TCZ	TWZ	KT	EW	IL
NS	1	0.198	0.272	0.540	0.208	0.074	0.478	0.316	0.108
SW	0.085	1	0.373	0.341	0.290	0.086	0.117	0.180	0.120
NG	0.059	0.090	1	0.160	0.102	0.270	0.240	0.286	0.126
WG	0.088	0.081	0.072	1	0.308	0.315	0.219	0.270	0.325
CZ	0.091	0.079	0.088	0.084	1	0.093	0.281	0.241	0.168
TWZ	0.095	0.148	0.086	0.108	0.131	1	0.372	0.199	0.090
KT	0.101	0.082	0.078	0.082	0.102	0.129	1	0.157	0.113
EW	0.083	0.062	0.051	0.067	0.088	0.107	0.031	1	0.400
IL	0.076	0.085	0.063	0.099	0.128	0.135	0.091	0.061	1

NS: North Shewa; SW: South Wollo; NG: North Gondar; WG: West Gojjam; TCZ: Tigray Central Zone; TWZ: Tigray West Zone; KT: Kembata Tembaro; EW: East Wollega; IL: Illubabor.

## Data Availability

The study was conducted at Holetta National Agricultural Biotechnology Research center molecular Laboratory.
